# Effects of a synbiotic as an antibiotic alternative on behavior, production performance, cecal microbial ecology, and jejunal histomorphology of broiler chickens under heat stress

**DOI:** 10.1371/journal.pone.0274179

**Published:** 2022-09-28

**Authors:** Ahmed Mohammed, Jiaying Hu, Raj Murugesan, Heng-Wei Cheng

**Affiliations:** 1 Faculty of Veterinary Medicine, Department of Animal and Poultry Behavior and Management, Assiut University, Assiut, Egypt; 2 Department of Animal Sciences, Purdue University, West Lafayette, IN, United States of America; 3 BIOMIN America, Inc., Overland Park, KS, United States of America; 4 USDA Agricultural Research Service, West Lafayette, IN, United States of America; University of Life Sciences in Lublin, POLAND

## Abstract

The aim of this study was to examine if synbiotics present similar efficiency to a common antibiotic used in poultry production under heat stress (HS) conditions. Two hundred and forty-one-day-old male Ross 708 broiler chicks were distributed among 3 treatments with 8 pens per treatment of 80 birds each for a 42-day trial. From day 15, birds were heat stressed (32°C for 9 h daily, HS) and fed the basal diet (CONT), the basal diet mixed with an antibiotic (Bactiracin Methylene Disalicylate) (0.05 g/kg of feed, BMD) or a synbiotic (0.5 g/kg of feed, SYN). The treatment effects on bird behavior, production performance, jejunal histomorphology, and cecal microbial ecology were examined. Behavioral observation was recorded by using instantaneous scan sampling technique. Production parameters were measured on day 14, 28, and 42. Cecal microbial populations of *Escherichia coli* and *Lactobacilli* and jejunal histomorphological parameters were measured at day 42. The results showed that, SYN birds exhibited more feeding and preening but less drinking and panting behaviors compared with both BMD and CONT birds (P < 0.05). The SYN birds also had higher body weight (BW) at both day 28 and 42 compared to CONT birds (P < 0.05). At the end of the experiment, the counts of *Escherichia coli* of SYN birds were at the similar levels of BMD but were lower than that of CONT birds (P < 0.05); while there were no treatment effects on the populations of *Lactobacilli* (P > 0.05). In addition, SYN birds had greater villus height compared with both CONT and BMD birds (P < 0.05). These findings suggest that the dietary synbiotic supplement has significant performance and welfare benefits, with the potential to be used as an alternative to antibiotics for poultry meat production, especially during hot seasons.

## Introduction

Heat stress (HS) is a critical animal health and welfare issue affecting the farm animal industries, impairing production performance and reducing economic profiles. Climate change over the recent decades has resulted in more hot days with more intense and frequent unexpected heat waves [1]. Heat stress results in annual economic losses of $1.69 - $2.36 billion in the livestock industry and of $128 - $165 million in the poultry industry [2]. In broiler chickens, especially during hot seasons in the tropical and subtropical regions, HS detrimentally affects production performances ranging from feed intake (FI), body weight gain (BWG), feed conversion ratio (FCR), and meat quality [3].

Broiler chickens, like other homeothermic animals, can maintain a relative body temperature by balancing the basal metabolic rate of body heat production and the rate of heat loss to the ambient environment [4]. However, broiler chickens are especially sensitive to HS due to poor heat tolerance and limited heat release by feathering, lacking sweat glands, and having a high metabolic rate [5, 6]. Behavioral adaptation is the major method of birds to cope with hot temperatures, including eating less and drinking more, seeking cooler areas, wing spreading (to promote cooling by reducing body insulation), and panting [7]. The act of panting increases blood partial pressure of carbon dioxide. If HS persist, excessive panting will cause birds to expire great amount of carbon dioxide and develop metabolic alkalosis, a serious disruption of acid-base balance, eventually leading birds to death [2].

The gastrointestinal tract (GIT) is one of the major organs affected by HS through several pathways, including organ ischemia and hypoxia, resulted from excessive panting and superficial vasodilation. In addition, HS negatively affects the function of the GIT in food digestion and nutrient resorption [8] and destroys microbial balance [9], causing local immunosuppression [10], damaging epithelial cells, and destroying the cellular microstructure and related intestinal barrier [11]. Consequently, HS disrupts intestinal homeostasis and increases gut permeability (leaky gut), leading to systemic inflammation and or infection [12].

Antibiotics have been used in broiler production for disease prevention since 1940s, with the secondary benefits, growth promotion, have also been observed [13]. In poultry, Bactiracin Methylene Disalicylate (BMD) has been used to control infectious diseases, subsequently improving growth performance and feed efficiency [14]. Bactiracin Methylene Disalicylate as an antibiotic act through interfering with cell wall production and protein synthesis to promote cell lysis [15]. However, the use of antibiotics in animal husbandry has caused growing public concerns about drug residues in meat products and the development of antibiotic-resistant bacteria [16]. In the United States, more than 2.8 million people get an antibiotic-resistant infection annually [17]. Therefore, the search for new alternatives in antimicrobial therapy in animal production has become critical. Attention is being paid particularly to natural product-based therapies, such as targeting the gut microbiota (or microbiome) with prebiotics, probiotics, and synbiotics [18, 19]. Synbiotics may be more efficient than prebiotics and probiotics as that synbiotics are a synergistic mixture of probiotics and prebiotics. Probiotics are live microorganisms whose functions in improving the microbiota balance in the GIT, inhibiting the growth of pathogenic bacteria, promoting food digestion and nutrient resorption, and boosting immune function [20]. Prebiotics are nondigestible fiber compounds that reduce the pathogenic bacteria through competing for binding sites on the intestinal mucosa and enhance the survival and growth of beneficial microbial species in the gut [21, 22]. Several studies have shown a promise outcome to use synbiotics as antibiotic replacements in commercial broiler chickens under thermoneutral temperatures [23–25], although some contrasting results have been reported [26, 27]. The inconsistent results could be affected by multiple factors such as the birds’ age and strain with different survivability, diet quantities and nutrients, and the format of synbiotics and dosage used between the different studies [28]. The objective of this study was to compare the effects of a synbiotic dietary supplement (a combination of fructo-oligosaccharides and 4 mixed microbial strains, SYN) and an antibiotic (Bactiracin Methylene Disalicylate, BMD) on the behavioral pattern, production performance, intestinal histomorphology, and cecal microbial ecology in heat stressed broiler chickens. Bactiracin Methylene Disalicylate as an antibiotic has been commonly used in poultry for promoting production and preventing and treating infectious diseases [29]. We hypothesize that SYN, similar to BMD, can mitigate the negative effects of HS on broiler health and welfare by preventing or reducing HS induced abnormal behaviors, impaired production performance, and damaged gut integrity without antibiotic side effects, producing antibiotic-resistant bacteria through improving the microbiota balance in the GIT, inhabiting HS-caused cell oxidative damage and related gut integrity disorder, increasing permeability (leaky gut), promoting food digestion and nutrient resorption, and reducing HS-caused immune suppression and related inflammation. we expect that the synbiotic has similar function as that antibiotic in improving behavior, performance production, microbial balance and intestinal histomorphology but without antibiotic side effects.

## Materials and methods

### Birds, management, and diets

A total of 240 one-day-old male broiler chicks (Ross708 strain) were obtained from a commercial hatchery (Pine Manor/Miller Poultry, Goshen, IN). The broiler chicks were weighed in groups of 10 birds each and assigned in to 1 of 24 floor pens (100×100 cm) with equal average body weight (BW) within the temperature-controlled room at the Poultry Research Farm of Purdue University. The pens were randomly distributed to one of three dietary treatments (n = 8): control group fed the basal diet (CONT) (Table 1), mixed with an antibiotic (Bactiracin Methylene Disalicylate, ZOETIS, Durham, NC, USA) at 0.05 g/kg (BMD) or a synbiotic (PoultryStar®, Biomin America Inc., Overland Park, KS, USA) at 0.5 g/kg (SYN). The dose was recommended by the company and tested in previous studies [3, 30]. The synbiotic supplement composed of fructooligosaccharides as the prebiotic and 4 microbial strains of probiotic (*Bifidobacterium animalis*; *Enterococcus faecium*; *Lactobacillus reuteri*; and *Pediococcus acidilactici*). The SYN and BMD were mixed separately into the basal diets based on bird life stages (the starter diet from day 1 to 14, grower diet from day 15 to 28, and finisher diet from day 29 to 42) by a step-up procedure until the total amount of diet was homogenously incorporated [3]. The study was performed during the summer of 2020. Broiler chicken management was performed according to the guidelines of Aviagen [31]. The ambient temperature was set at 34 ± 2°C on day 1 and was reduced 3°C per week until it reached 26 ± 2°C on day 14; thereafter, HS, 32°C for 9 h (0800–1700), was started daily on day 15 (i.e., the beginning of the growth phase) and maintained until the end of the experiment. In this study, we did not use a thermoneutral control group as the objective of the study was to investigate the effect of synbiotics an antibiotics alternative on HS birds and using the daily HS episode (32°C/9 h/d) was guaranteed to motivate HS based on previous reports [19, 32, 33]. Narrowing focus to involve only HS broiler chickens allowed us to minimize animal use by 50%, a key priority of animal welfare scientists (i.e., the 3Rs principal [3, 19, 34]). The experimental protocol and related animal treatment and care procedures were approved by the Animal Care and Use Committee of Purdue University (West Lafayette, IN, USA) (PACUC number:1712001657).

**Table 1:** Components of base diet ^1^, separated by the growth phase^2^.

Ingredient %	Starter	Grower	Finisher
	(1–14 day)	(15–28 day)	(29–42 day)
**Corn ground**	57.66	63.76	66.9
**Soybean meal 47.5%**	35.27	29.68	26.3
**Soybean oil degummed**	3	3	3.52
**Calcium carbonate**	1.41	1.38	1.49
**Phosphate monocalcium**	1.42	1.02	0.82
**L-Lysine**	0.11	0.1	0.02
**Salt plain**	0.48	0.46	0.48
**L-Threonine 98%**	0.06	0.04	0
**DL-Methionine**	0.24	0.21	0.12
**Poultry turkey starter**	0.35	0.35	0.35
Calculated Analysis ^3^			
**Crude protein %**	23.4	22.8	19.2
**Poultry ME kcal/kg**	3050	3151	3200
**Calcium %**	0.95	0.85	0.75
**Available phosphorus %**	0.50	0.44	0.36
**Methionine %**	0.66	0.59	0.53
**Methionine+Cystine %**	1.04	0.97	0.86
**Lysine %**	1.42	1.29	1.09
**Threonine %**	0.97	0.89	0.74
Na %	0.22	0.20	0.19

^1^ The ration formulation was produced according to Aviagen [31]. Dietary treatments containing basal diet under heat stress condition (CONT), mixed with an antibiotic Bactiracin Methylene Disalicylate (BMD) and a synbiotic (SYN).

^2^ The diets were formulated by the Purdue University Feed Mill. (w. Lafayette, IN, USA).

^3^ Provided per kilogram of diet: vitamin A, 13.233 IU; vitamin D3, 6.636 IU; vitamin E, 44.1 IU; vitamin K, 4.5 mg; thiamine, 2.21 mg; riboflavin, 6.6 mg; pantothenic acid, 24.3 mg; niacin, 88.2 mg; pyridoxine, 3.31 mg; folic acid, 1.10 mg; biotin, 0.33 mg; vitamin B12, 24.8 μg; choline, 669.8 mg; iron from ferrous sulfate, 50.1 mg; copper from copper sulfate, 7.7 mg; manganese from manganese oxide, 125.1 mg; zinc from zinc oxide, 125.1 mg; iodine from ethylene diaminedihydroidide, 2.10 mg; selenium from sodium selenite, 0.30 mg.

### Behavioral observations

Forty birds per treatment (5 birds per pen x 8 pens per treatment) were randomly selected for behavioral observation and marked with a livestock green spray paint marker (Cotran Corporation, Portsmouth, RI). Behavioral observations were performed twice daily, from 09:00 to 10:00 and 13:00 to 14:00, for 3 days weekly (Sunday, Tuesday, and Thursday) from week 3 to 6. Instantaneous scan sampling was used for recording the focal birds’ behaviors 6 times per observation session based on the developed ethogram (Table 2) [3]. There was 1 m distance between the observer and each pen during the behavioral observation to avoid disturbing birds’ behavior. Behavioral data are presented as: % of behavior = the number of a behavior / the total number of all behaviors during the observation time [3, 35].

**Table 2:** Ethogram of broiler behaviors according to Mohammed et al. [3].

Behavior^1^	Definition
**Standing**	**The birds’ body posture is in an upright position. The feet are in touch with the litter. No other body part is in contact with the floor surface.**
**Sitting**	**The ventral part of the bird is touching the ground. Legs are bent at the knee with lower part of the leg, under the knee (i.e., fibula and tibia) touching the ground.**
**Feeding**	**The bird’s head is in the feeder, presumably eating feed.**
**Drinking**	**The bird’s neck is stretched to place his beak towards the drinker and then moved up, presumably drinking water.**
**Preening**	**The bird is using the beak to manipulate its own feathers gently.**
**Wing Spreading**	**Wings are extended horizontally from the body such that a space can be seen between the underside of the wing and the surface of the bird’s body.**
**Panting**	**The bird opens its beak to breathe, and respiration rate is abnormally fast.**

^1^All behavioral patterns were alternatively exclusive; postures (i.e., standing and sitting) were only enumerated if the bird did no other simultaneous behaviors.

### Growth performance

Production parameters (BW, BWG, FI, and FCR) were measured at the end of each growth phase, day 14 (the end of starter phase), day 28 (the end of grower phase), and day 42 (the end of finisher phase), according to Mohammed et al. [3]. All birds within a pen (10 birds per pen) were weighted at each time point. Feed intake was calculated by subtracting residual feed from the offered feed. The BWG was calculated as the BW of the present time point subtracted the BW of the previous time point. Data of FI and BWG were used to calculate the FCR (FCR = FI/BWG).

### Sample collection

At the end of the experiment, one bird per pen was randomly chosen for sample collection (8 birds per treatment). The sampled birds were sedated using sodium pentobarbital (30 mg/mL) for blood sample collection. Followed blood collection, the birds were killed by cervical dislocation, and then jejunal samples (2 cm at the midpoint) [36] and cecal contents (1 gram) were collected [37]. The cecal samples were stored at -80°C until analysis. The jejunal tissue samples were gently flushed with 0.9% saline to remove the contents and then fixed in 10% formalin until analysis.

### Histomorphological measurements

The histomorphologal parameters of the jejunal tissue samples were measured using previously published methods [34, 38]. Briefly, the jejunal tissue samples were dehydrated and embedded with paraffin wax (Thermo fisher scientific, Kalamazoo, MC). The paraffin blocks were cut at 5-μm-thick cross sections using a microtome, then stained with hematoxylin and eosin (H&E) (GeneCopoeia, Rockville, MD), and examined under an Olympus BX40 F-3 microscope (Olympus Cooperation, Tokyo, Japan) attached to a digital video camera (Q- imaging, 01-MBF-200R-CLR-12, SN: Q32316, Canada) as described in Jiang et al. [34]. The morphometric measurements of villus height and crypt depth of the jejunum were analyzed by using the software of Image J (National Institutes of Health, USA).

### Microbial analysis

Bacterial enumeration of each cecal sample was performed by following the previously published protocol [37, 39]. In brief, 1 g of cecal sample was mixed with 9 mL of buffered peptone water (NeogenCorporation, Lansing, MI), and then 10-fold serial dilutions up to 10^−7^ were prepared. A 10 μL sample mixture from each of the serial dilutions was inoculated using bacteria-specific agars. Rogosa agar (Fisher Scientific/Becton, Dickinson Co.) was used and incubated for 24 h at 37°C anerobically for enumeration of total *lactobacilli*; and Eosin methylene blue (EMB) agar (Fisher Scientific/Becton, Dickin-son Co., Sparks, MD) was used and incubated for 24 h at 37°C aerobically for enumeration of *Escherichia coli*. Counting of the colonies was done as units per gram of the sample after incubation.

### Statistical analysis

The experiment was conducted in a randomized block design. A pen (n = 8) was considered as the experimental unit. Behavioral patterns and growth performance parameters were analyzed by repeated measures ANOVA, and the cecal microbial population and jejunal histomorphological parameters were analyzed by One Way Analysis of Variance. The data were analyzed by using PROC MIXED model with SAS 9.4 software (SAS Institute Inc., Cary, NC). The Shapiro-Wilk test was used to analyze the normality of the data. Transformation of the data was performed for normality when variances were not homogeneous [40]. Performance parameters and behavioral patterns were log transformed. Because statistical trends were similar for both transformed and untransformed data, the untransformed results were presented. For intestinal bacterial colony forming units (CFUs), colony counts (cfu/g) of *Escherichia coli* and *lactobacillus* spp., were exposed to logarithmic transformation (log10) for normality and the transformed data were presented. Tukey-Kramer test was used to compare the means when a significant difference was detected; the level of statistical significance was set when the coefficients were at P < 0.05. Data were presented as mean ± SE.

## Results

### Behavioral patterns

The effects of dietary supplementation of BMD and SYN on behavioral patterns of heat stressed broiler chickens are presented in Table 3. Compared to CONT and BMD groups, the SYN group, exhibited more standing (P < 0.05), feeding (P < 0.001), and preening (P < 0.001), with less drinking (P < 0.01) and panting (P < 0.001). In addition, the SYN group sat less than the CONT group (P < 0.05) but not the BMD group (P > 0.05), while wing spreading in the SYN group was not different compared to both CONT and BMD groups (P > 0.05). There were no treatment effects on all measured behaviors between BMD and CONT groups (P > 0.05) except the sitting behavior (P < 0.05).

**Table 3:** Effect of dietary supplementation of synbiotic (SYN) and antibiotic (BMD) on behavioral patterns of broiler chickens reared under heat stress condition.

Behavior	CONT	BMD	SYN	*P*-value
**Standing (%)**	3.81±1.09^b^	3.27±1.09^b^	7.92±1.09^a^	0.0128
**Sitting (%)**	30.89±2.35^a^	20.46±2.35^b^	15.21±2.35^b^	0.0004
**Feeding (%)**	18.51±1.74^b^	17.53±1.36^b^	33.00±1.75^a^	0.0001
**Drinking (%)**	6.24±0.88^a^	6.76±0.68^a^	3.12±0.54^b^	0.0033
**Preening (%)**	0.75±0.24^b^	1.59±0.35^b^	4.45±0.62^a^	0.0001
**Wing Spreading (%)**	6.13±0.68	5.97±0.98	3.58±1.01	0.1045
**Panting (%)**	49.30±2.87^a^	44.36±3.58^a^	16.95±2.23^b^	0.0001

^a,b^Mean± SE with different superscripts in the same row differ (*P* < 0.05). (n-8 per treatment; and the data were collected from 40 birds/treatment; 5 birds/pen x 8 pens/treatment).

^1^CONT, heat stress + a basal diet; BMD, heat stress + the basal diet mixed antibiotic Bactiracin Methylene Disalicylate; SYN, heat stress + the basal diet mixed synbiotic.

### Growth performance

The effects of dietary supplementation of BMD and SYN on growth performance parameters of heat stressed broiler chickens are presented in Table 4. At day 14, there were no treatment effects on all production traits (P > 0.05) except FI was increased in the CONT group compared to BMD group but not SYN group. At day 28, compared to the CONT group, the SYN group had greater BW, BWG, but lower FCR (P *<* 0.05) without difference in FI (P > 0.05); while BMD groups had a lower FI and FCR (P *<* 0.05) without differences in the BW and BWG. Feed intake (P *<* 0.05) was the only difference between SYN and BMD groups, the former had higher FI (P *<* 0.05). At day 42, among measured production traits, the SYN group had greater BW and BWG (P < 0.05) than CONT, but not BMD groups. While FI and FCR in the SYN group were not different compared to both CONT and BMD groups (P > 0.05). There were no treatment effects on all measured parameters between SYN and BMD groups (P > 0.05).

**Table 4:** Effect of dietary supplementation of synbiotic (SYN) and antibiotic (BMD) on performance parameters of broiler chickens reared under heat stress condition.

Treatment^1^	CONT	BMD	SYN	*P*-value
**d14**				
**BW (g)**	432.28±7.35	428.62±4.99	445.79±6.72	0.1562
**FI (g)**	432.75±10.77^a^	390.83±8.09^b^	408.29±13.25^a^^b^	0.0414
**BWG (g)**	395.94±7.37	395.75±6.20	409.49±6.72	0.2815
**FCR**	1.09±0.01	1.01±0.02	1.00±0.04	0.0581
**d28**				
**BW (g)**	1396.01±16.68^b^	1425.20±25.88^a^^b^	1497.15±13.88^a^	0.0075
**FI (g)**	1527.62±34.94^a^	1368.12±42.77^b^	1520.00±18.32^a^	0.0073
**BWG (g)**	963.73±12.82^b^	997.21±22.83^a^^b^	1051.36±18.73^a^	0.0173
**FCR**	1.58±0.02^a^	1.37±0.03^b^	1.45±0.03^b^	0.0007
**d42**				
**BW (g)**	2380.97±25.53^b^	2434.16±28.33 ^a^^b^	2512.34±37.06^a^	0.0325
**FI (g)**	2093.72±25.41	2043.66±23.15	2119.30±24.37	0.1371
**BWG (g)**	997.47±22.52 ^b^	1042.31±12.99^a^^b^	1061.47±11.05 ^a^	0.0471
**FCR**	2.11±0.04	1.96±0.03	1.99±0.02	0.0521

^a,b^Mean± SE with different superscripts in the same row differ (*P* < 0.05). (n-8 per treatment; and the data were collected from 80 birds/treatment; 10 birds/pen x 8 pens/treatment).

^1^CONT, heat stress + a basal diet; BMD, heat stress + the basal diet mixed antibiotic Bactiracin Methylene Disalicylate; SYN, heat stress + the basal diet mixed synbiotic.

### Cecal microbiota

The effects of dietary supplementation of BMD and SYN on the cecal populations of *Escherichia coli* and *Lactobacillus* spp. in heat stressed broiler chickens are presented in Table 5. Heat stress had significant effects on the cecal population of *Escherichia coli* as that CONT had the highest counts at day 42 (P < 0.05). The HS effects were reduced by dietary supplements, but significant reduction found in the SYN group only (P < 0.05). There was no treatment effect of dietary treatments on *Lactobacillus* spp. count of heat stressed broiler chickens (P > 0.05).

**Table 5:** Effect of dietary supplementation of synbiotic (SYN) and antibiotic (BMD) on cecal bacterial populations (*Escherichia coli* and *Lactobacilli*) of broiler chickens reared under heat stress condition.

Treatment^1^	CONT	BMD	SYN	*P*-value
***Escherichia coli***	3.12±0.12^a^	2.83±0.08^a^^b^	2.48±0.15^b^	0.0061
***Lactobacilli***	2.55±0.21	2.52±0.22	2.95±0.15	0.2521

^a,b^Mean± SE with different superscripts in the same row differ (*P* < 0.05). (n-8 per treatment; and the data were collected from 8 birds/treatment; 1 birds/pen x 8 pens/treatment).

^1^CONT, heat stress + a basal diet; BMD, heat stress + the basal diet mixed antibiotic Bactiracin Methylene Disalicylate; SYN, heat stress + the basal diet mixed synbiotic.

### Histomorphological measurements

**Fig 1:**
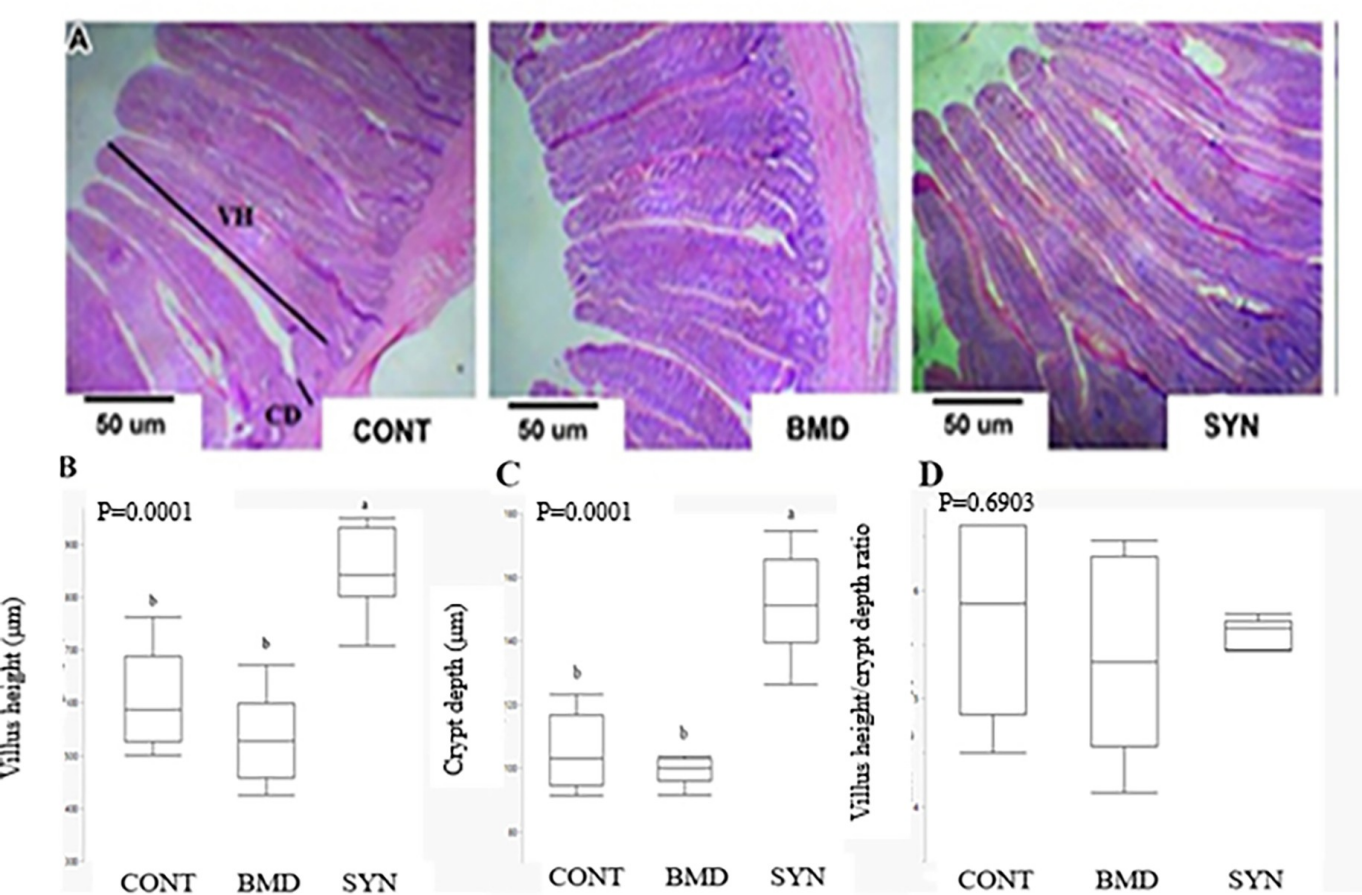
The examples of the morphological changes of the villus height and crypts depth in the jejunum of broiler chickens. Treatments: Heat stressed birds fed the basal diets (CONT), mixed antibiotic Bactiracin Methylene Disalicylate (BMD) or Synbiotic (SYN). VH: Villus height; CD: Crypt depth. The scale bar = 50 um.

The dietary supplementation effects on villus height, crypt depth and the ratio of villus height and crypt depth in the jejunum of heat stressed broiler chickens are presented in Fig 1. The SYN group had longer villi (P < 0.01) and crypts depth (P < 0.01) than both BMD and CONT groups. These changes resulted in the greatest resorption areas in the SYN group (villus height + crypts depth: SYN, 1001 μm ≥ CONT, 711 μm ≥ BMD, 632 μm). There was no treatment effect of dietary treatments on the ratio of villus height and crypt depth of heat stressed broiler chickens (P > 0.05). There were no treatment effects on measured histomorphological parameters (P > 0.05) between BMD and CONT groups.

## Discussion

Heat stress is among the most harmful environmental stressors for the poultry industry due to it significant damages to health and welfare of birds which could lead to economic losses [2–41]. When exposed to an environmental temperature exceeding the upper critical limit of their thermoneutral zone, birds maintain their relative body temperature attempting to cope with the health risk by losing body heat through physiological, biochemical, and behavioral changes [42, 43]. If heat persists, excessive reactions beyond birds’ adaptive capability may cause pathophysiological disorders, eventually increasing mortality. Sub-therapeutic doses of antibiotics have been used as growth promoters to improve production performance and to prevent various diseases in broiler chickens. However, ban and severe restriction on use of antibiotics have been practiced in most countries due to growing concerns about antibiotic residue in poultry products and related food safety. Previous studies have evidenced that keeping a healthy gut microbiome is a critical step to maintain effective food digestion and nutrient resorption, contributing to local and systemic immunity [22–37]. The current results indicate that the dietary synbiotic supplement not only reduced HS-induced behavioral changes, particularly, panting and drinking, but also increased production performance, especially BW in broiler chickens. In addition, the synbiotic supplement increased the jejunal villus height and reduced cecal *Escherichia coli* count.

Under high environmental temperatures, birds minimize their feed consumption as a mechanism to reduce body metabolism and related heat production [2–39]. As a result, reduced FI and production performance (meat production and carcass quality) and increased mortality have been observed in broilers [4]. Compared to CONT group, SYN birds performed more feeding, and preening, but less HS-related behavior (panting and drinking). Also, SYN group had higher BW and BWG at the end of both the grower and finisher phases. Additionally, SYN group had a longer jejunal villi with a lower count of cecal *Escherichia coli*. These results may indicate that the synbiotic supplement functionally increases gut resorption surface and reduces gut pathogenic bacteria. In agreement with our findings, several studies also reported synbiotics maintain or improve broiler production, health, and welfare profiles under both thermoneutral and HS conditions [3, 24, 30]. However, the outcomes are not consistent; some studies have reported that dietary supplementation of probiotics and synbiotics had no significant effect on BWG, FI, FCR, intestinal morphology, and bacterial populations [44, 45]. The different findings could be affected by multiple factors such as the birds’ strains, age, and health status; diet quantity and nutrient content; housing environment and ambient temperature (i.e., the severity and length of HS); and the format of synbiotic and its dosage used in the different studies. The current synbiotic consists of fructooligosaccharides as the prebiotic and four microbial strains of probiotic bacteria extracted from chicken GIT. It may have more species-specific efficiency in chickens than other probiotic bacteria extracted from other animals.

Numerous studies have reported that probiotics can regulate birds’ health and welfare via the functions in immunomodulation [46], neuroendocrine regulation [47], and nutrient metabolism [48]. In addition, prebiotics can stimulate the growth and activity of beneficial bacteria in the GIT [3]. Probiotic and prebiotic of a synbiotic may work together (forming a synergism) to improve animals’ health through regulation of both the microbiota-gut-immune axis [49] and the microbiota-gut-brain axis [50]. For example, Hassan et al. [51] revealed an increase in hematocrit values, erythrocyte count and hemoglobin concentration in broiler chickens supplemented with *lactobacillus* spp. Improvement in the HS-related behaviors in the synbiotic supplemented birds reported here may be attributed to the similar changes of hematological parameters. In one of our previous studies, the synbiotic supplement reduced heterophil/lymphocyte ratio, a stress indicator, without effects on the levels of circulating monocytes, eosinophils, and basophils in HS broilers [37]. For explanation of the effects of increased preening in the SYN group, Mohammed et al. [3] noted an improvement in preening behavior of broiler chickens supplemented with synbiotics under heat stress condition. In chickens, preening has been thought to be a comfort-related behavior [52], and it’s likely that supplementing broiler chickens with synbiotics reduced the disturbance associated with HS.

The protective effect of synbiotics on the bird intestinal microbial ecology under elevated temperature conditions was revealed in this study. The improvement in the jejunal histomorphological characters may be attributed to the functions of both prebiotic nondigestible fibers and probiotic beneficial bacteria in protecting the jejunal villi from pathogens and toxins to strengthen gut integrity (the tight junctions and epithelial cell cytoskeletons) through improved nutrient metabolism and absorption [53]. Similarly, one of our previous studies has reported the improvement in the intestinal architecture in synbiotic fed broilers under HS conditions [34]. Hassanpour et al. [54] also reported a synbiotic composed of probiotic *Enterococcus faecium* (DSM 3530 strain) and prebiotic fructooligosaccharides, phycophytic substances derived from sea algae and cell wall fragments, enhanced intestinal health of broilers under thermoneutral conditions.

Antibiotics have been utilized as growth promoters in poultry meat production for many years. The antibiotics mechanism of action against various infectious diseases is related to interactions with intestinal microbial population [13]. Compared to CONT group, BMD group performed less sitting behavior, and there was no significant difference in the count of cecal *Escherichia coli*. In this study, the SYN and BMD groups had a similar effect on the production performance parameters such as BW, BWG, and FCR as well as cecal microbial count of *Escherichia coli* and villus height/crypts depth ratio, but SYN group had lower HS-related behaviors and longer villus height and crypts depth than BMD group, resulting in a greater resorption area. It may indicate that the SYN has more benefits to the gut homeostasis than BMD. Synbiotics have functions in protecting intestinal epithelial cells and maintaining gut microstructural integrity due to antimicrobial, anti-inflammatory, antioxidant, enzymatic, and immunomodulatory activities [34–55]. In agreement with the hypothesis, Neveling and Dicks [56] reported that a probiotic (combination of *Enterococcus faecium*, *Pediococcus acidilactici*, *Bacillus animalis*, *Lactobacillus salvarius*, *and Lactobacillus reuteri)* reduced the colonization of pathogens in the GIT of broilers. Gutierrez-Fuentes et al. [57] also reported a probiotic (consisting of *Lactobacillus salivarius* and *Pediococcus parvulus*) improved broiler weight gain, bone health, intestinal integrity, and immunity. Interestingly, SYN and BMD exhibited different effects on behavioral patterns. The SYN birds displayed more feeding and preening but less panting and drinking. These results may indicate the synbiotic has functions in promoting growth and health in broilers exposed to HS, which is better or, at least, similar to the function of BMD. In supporting our findings, several studies have shown a promise in terms of protecting the broiler profiles when supplementing with synbiotics instead of antibiotics [24, 30]. The mechanisms underlying the different effects between synbiotics and antibiotics were not detected in this study but could be similar to the ones reported previously. It has been proposed that antibiotics, as growth promotors, prevent gut infectious disease but kill all gut bacteria (including both beneficial and pathogenic bacteria), while synbiotics as well as prebiotics and probiotics, as growth promotors, eliminate pathogenic bacteria but improve beneficial bacteria. In addition, synbiotics have multiple functions in food digestion, nutrient and mineral resorption, immunomodulation, and release of bioactive factors [58–60]. Furthermore, synbiotics may regulate HS response and related local and systemic inflammation through the bidirectional communication between the GIT and brain via the microbiota-gut-brain axis [19–61]. Regulation of the microbiota-brain-gut axis, especially the hypothalamic-pituitary-adrenal system, is essential for maintaining an animal’s physiological and behavioral homeostasis. Taken together, the synbiotic may inhibit or reduce negative effects of HS on the GIT by restoring the functions of gut microbiota; improving gut immunity [62]; increasing gut integrity to reduce intestinal barrier permeability [63, 64]; and or assisting with beneficial bacteria’s functions in nutritional benefits [65].

## Conclusion

The current results indicate that the beneficial effects of the SYN supplement on broiler production are better or, at least, comparable with BMD. The SYN reduced the negative effects of HS on production performance with increased the jejunal villus height and crypts depth. The SYN also reduced HS-associated behaviors including panting and drinking. The current results suggest that dietary synbiotics could be a useful management strategy for replacing antibiotics to improve chicken health, welfare, and production during hot seasons, especially in the tropical and subtropical regions.

## Supporting information

S1 Data (XLSX)
